# The chromosomal association/dissociation of the chromatin insulator protein Cp190 of *Drosophila melanogaster *is mediated by the BTB/POZ domain and two acidic regions

**DOI:** 10.1186/1471-2121-11-101

**Published:** 2010-12-31

**Authors:** Daniel Oliver, Brian Sheehan, Heather South, Omar Akbari, Chi-Yun Pai

**Affiliations:** 1 University of Nevada, Reno, Biology Department, NV 89557, USA

## Abstract

**Background:**

Chromatin insulators or boundary elements are a class of functional elements in the eukaryotic genome. They regulate gene transcription by interfering with promoter-enhancer communication. The Cp190 protein of *Drosophila melanogaster *is essential to the function of at least three-types of chromatin insulator complexes organized by Su(Hw), CTCF and BEAF32.

**Results:**

We mapped functional regions of Cp190 in vivo and identified three domains that are essential for the insulator function and for the viability of flies: the BTB/POZ domain, an aspartic acid-rich (D-rich) region and a C-terminal glutamic acid-rich (E-rich) region. Other domains including the centrosomal targeting domain and the zinc fingers are dispensable. The N-terminal CP190BTB-D fragment containing the BTB/POZ domain and the D-rich region is sufficient to mediate association with all three types of insulator complexes. The fragment however is not sufficient for insulator activity or viability. The Cp190 and CP190BTB-D are regulated differently in cells treated with heat-shock. The Cp190 dissociated from chromosomes during heat-shock, indicating that dissociation of Cp190 with chromosomes can be regulated. In contrast, the CP190BTB-D fragment didn't dissociate from chromosomes in the same heat-shocked condition, suggesting that the deleted C-terminal regions have a role in regulating the dissociation of Cp190 with chromosomes.

**Conclusions:**

The N-terminal fragment of Cp190 containing the BTB/POZ domain and the D-rich region mediates association of Cp190 with all three types of insulator complexes and that the E-rich region of Cp190 is required for dissociation of Cp190 from chromosomes during heat-shock. The heat-shock-induced dissociation is strong evidence indicating that dissociation of the essential insulator protein Cp190 from chromosomes is regulated. Our results provide a mechanism through which activities of an insulator can be modulated by internal and external cues.

## Background

Chromatin in the eukaryotic cell nucleus is organized into sub-regions of various transcriptional activities. Chromatin insulators, also known as boundary elements, are a unique class of functional elements in eukaryotic genomes. They are thought to separate differently regulated sub-regions along chromatin fibers. Deletion of an insulator can cause abnormal expression of local genes resulting in developmental defects. For example, deletion of the Fab-7 insulator in Bithorax complex of *Drosophila melanogaster *results in body segment transformation [1].

Chromatin insulators interfere with promoter-enhancer interactions only when they are positioned between a promoter and the enhancer. The *gypsy *insulator of *Drosophila melanogaster *is one of the best characterized insulators. Insertion of a copy of the *gypsy *insulator sequence in a gene or its regulatory region interferes with interactions between local enhancers and the promoter thus causing mutant phenotypes in many genes [2,3]. The *gypsy *insulator is a 340 to 430 base pair sequence containing 8 or 12 copies of a consensus repeat sequence, some of which bind the Suppressor of Hairy-wing [Su(Hw)] zinc finger protein, which is required for insulator activity [2,4-6].

Su(Hw) organizes a protein complex on the *gypsy *insulator. Identified proteins in the complex include Su(Hw), the Centrosomal Protein 190 (Cp190), Modifier of mdg 4 67.2 [Mod(mdg 4)67.2], and several other proteins [7-13]. The Cp190 protein is essential for *gypsy *insulator function too [11] and is present in other types of chromatin insulator complexes such as the CTCF complex which mediates the insulator activity at the Fab-8 insulator in the Bithorax complex [14-16], and the BEAF32 complex [16,17].

Cp190 has three conserved protein motifs: (1) The Broad-complex, Tramtrack and Bric-abrac (BTB) homologous domain, also know as the Poxvirus and Zinc Finger (POZ) domain; (2) three copies of C2H2 zinc fingers; and (3) the C-terminal E-rich domain. In addition to these three domains, previous studies identified a centrosomal targeting domain (CENT) for localizing the Cp190 protein to centrosomes during mitosis [18]. To understand the roles of these domains in insulator function, we used genetic complementation using P-element transgenes expressing domain-truncated Cp190 mutants. We identified an additional acidic D-rich region which is involved in the association of Cp190 with insulator complexes. We found that the BTB domain, the D-rich region and an acidic C-terminal E-rich region are essential to the function of Cp190 in the *gypsy *insulator. The zinc fingers and the centrosomal targeting domain are dispensable. Our results indicate that the three essential domains have distinct roles in insulator binding and function.

## Results

### Cp190 domain-truncated mutants

**Figure 1:**
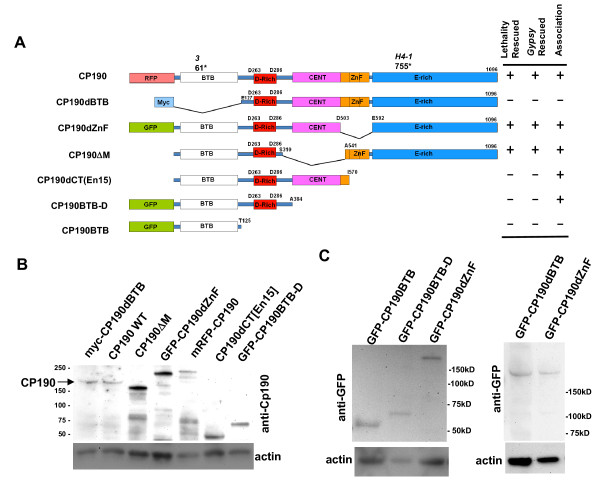
**Protein structure and expression levels of *CP190 *deletion mutants**. (A) Schematic diagram of *CP190 *deletion mutants and their genetic complementation phenotypes. The full-length Cp190 is tagged with mRFP (mRFP-CP190). Each *CP190 *mutant contains a deletion of one of the functional domains. CP190dBTB lacks the BTB domain and is tagged with myc (myc-CP190dBTB) or with GFP (GFP-CP190dBTB, not shown); CP190dZnF lacks all three zinc fingers and is tagged with GFP (GFP-CP190dZnF); CP190ΔM lacks the centrosomal targeting domain (CENT); CP190BTB-D lacks CENT, zinc fingers and the E-rich domain and is tagged with GFP (GFP-CP190BTB-D); CP190BTB has only the BTB domain and is tagged with GFP (GFP-CP190BTB). The CP190dCT(En15) is the predicted protein from the EMS-induced *CP190*^*En15 *^mutant, which lacks two zinc fingers and the E-rich region. The amino acids at the junction of each deletion are indicated. (B-C) Expression of the mutated Cp190 proteins revealed by the anti-CP190 immunoblot (B) or by anti-GFP immunoblots (C). All transgenic lines were crossed into the homozygous *CP190*^*3 *^background. Proteins extracted from about two 3^rd ^instar larvae containing the indicated transgene were loaded per each lane. Similar results were also obtained from pupae 24-48 hours after pupation. Underneath the blots were stripped filters re-probed with the anti-actin antibody as a loading control.

To determine functional domains essential for the function of Cp190 in the *gypsy *chromatin insulator, we performed genetic complementation with P-element transgenes carrying CP190 mutants, each lacking a predicted functional domain (Figure 1A). Since *CP190 *is expressed ubiquitously in cells of all examined tissues in all developmental stages and that *CP190 *mutations were rescued by a *CP190 *cDNA driven by the *Ubiquitin Ubi63e *promoter [19], we expressed Cp190 proteins using the P-element vectors containing the *Ubiquitin *Ubi63e promoter [20]. Each P-element transgene contains a full-length or a mutated *CP190 *cDNA fragment fused to either the green fluorescent protein (GFP), the red fluorescent protein (RFP) or a 6x-Myc tag (Figure 1A). The molecular tags allow detection of the transgenic fusion proteins by anti-tag antibodies or by GFP or RFP fluorescence. At least two independent insertions of each P-element were crossed into homozygous *CP190 *mutant backgrounds. These include CP190^3 ^nucleotide substitution (C368T, NM79635.1) that causes a translation stop at Q61 and is homozygous lethal in the pupal stage [19,21], and *CP190*^*H4-1*^, a viable mutant encoding the N-terminal 755 amino acids [11]. Homozygous *CP190*^*3 *^larvae do not express detectable amounts of the predicted truncated protein and thus are essentially null mutants. In addition to the transgenes, we also included the *CP190*^*En15*^, which is not a transgene but an ethyl methanesulfonate (EMS) generated mutant, in this series of domain-truncation analysis. *CP190*^*En15 *^is a point mutation (C1889G, NM079635.1) that causes a stop codon after the amino acid residue 570 (Y571*). The *CP190*^*En15 *^mutant expresses a truncated protein marked as CP190dCT(En15) in Figure 1, which lacks the whole C-terminal E-rich region and two of the zinc fingers (see below for more details).

### The Cp190 BTB domain, but not the zinc finger or centrosomal targeting (CENT) domains, is required for viability and insulator activity

Expression of engineered Cp190 truncations were examined by immunoblots using lysates from homozygous *CP190*^*3 *^flies carrying the transgenes at larval stages (Figure 1B and 1C) or pupal stages (not shown) using anti-Cp190 (Figure 1B) or anti-GFP immunoblots (Figure 1C). Similar results were obtained from both larvae and pupae. The expected truncated proteins were expressed at levels similar to, or higher than, the wild-type Cp190. Smaller degraded fragments were noticeable in CP190ΔM, GFP-CP190dZnF and mRFP-CP190 transgenic lines.

We next determined if the transgenes rescue the lethality of homozygous *CP190*^*3*^. Expression of mRFP-CP190 encoded by *P[Ubi63e::mRFP-CP190, w*^*+*^], or GFP-CP190dZnF lacking all three zinc fingers encoded by *P[Ubi63e::GFP*-CP190dZnF, mini-w^*+*^], fully rescued the lethality of homozygous CP190^3^. The rescued adults were healthy and fertile, showing that the GFP-CP190dZnF and the mRFP-CP190 proteins support all essential Cp190 functions. We confirmed the published result that the CP190ΔM transgene which lacks the centrosomal-targeting CENT region (Figure 1A) rescues lethality of the homozygous *CP190*^*3 *^mutant [19].

**Figure 2:**
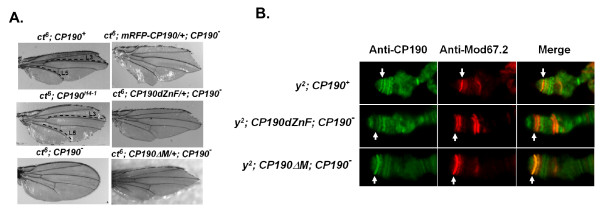
**The GFP-CP190dZnF and the Cp190ΔM proteins are fully functional in the *gypsy *insulator**. (A) The *gypsy *insulator-dependent wing phenotype of the *ct*^*6 *^mutation in the indicated genetic backgrounds. Wings of the *ct*^*6 *^flies have a cut shape and lack of margin bristles (top left). The two doted lines mark the veins L3 and L5 between which the margin bristles are not developed at all. The *ct*^*6 *^; CP190^H4-1 ^wing has some margin bristles between L3 and L5, and is rounder in shape (middle left). The wing shape of the *CP190 *deficient *CP190*^*3*^*/CP190*^*P1 *^flies is restored to a *ct*^*+*^-like shape with fully developed margin bristles (bottom left), indicated. A copy of the *P[Ubi63e::mRFP-CP190] *(top right), *P[Ubi63e::GFP-CP190dZnF] *(middle right) or the *P[CP190ΔM] *(bottom right) transgene rescues the defective *gypsy *insulator function in the *CP190 *deficient *CP190*^*3*^*/CP190*^*P1 *^background. The rescued flies have the cut wing shape similar to the *ct*^*6*^*; CP190*^*+ *^flies. (B) Localization of the mutated Cp190 proteins to the *gypsy *insulator insertion at the *y *locus on the *y*^*2 *^polytene chromosome. Shown are the tips of X chromosomes. The *y *locus that contains a copy of the *gypsy *insulator is indicated by white arrows. Distribution of the GFP-CP190dZnF protein (middle panel) and the Cp190ΔM protein (bottom panel) on the polytene chromosomes of the indicated flies was revealed by anti-Cp190 (left column). The anti-Mod(mdg4)67.2 (middle column) shows the distribution of the Su(Hw) insulator complex. The right column shows the merged images of the left and the middle columns.

The zinc finger and centrosomal targeting domains are also not required for *gypsy *insulator activity. The insulator function was evaluated using two *gypsy *insertion mutations that cause adult phenotypes: the cut wing phenotype of the *ct*^*6 *^mutation (Figure 2A) and the body cuticle pigmentation phenotype of the y^2 ^mutation. ct^6 ^wing margins lack bristle cells (Figure 2A top left). The *ct*^*6 *^margin phenotype is suppressed in a CP190-deficient background. For example, in the homozygous viable CP190^H4-1 ^flies, some margin bristles appear between veins L3 and L5 and wings are rounder in shape (Figure 2A, middle left), indicating that the *gypsy *insulator activity is partially reduced. Most CP190^3^/CP190^P1 ^flies die as pupae, but a few adult escapers (2%) have wings with wild-type appearance (Figure 2A, bottom left), indicating greatly reduced insulator activity. A copy of mRFP-CP190, GFP-CP190dZnF or the CP190ΔM transgenes restored the *gypsy *insulator function in the homozygous CP190^3^, which cause substantial wing margin loss (Figure 2A, right column).

In contrast, the BTB domain of Cp190 is required for viability and insulator activity. Neither the myc-tagged myc-CP190dBTB encoded by P[*Ubi63e::myc-CP190dBTB, mini-w*^*+*^], nor the GFP-tagged GFP-CP190dBTB encoded by P[*Ubi63e::GFP-CP190dBTB, mini-w*^*+*^], rescue the lethality of homozygous *CP190*^*3 *^although they were expressed at substantial levels (Figure 1). To evaluate if the myc- or GFP-CP190dBTB transgenes rescue the defective *gyspy *insulator function, the transgenes were crossed into the homozygous viable *CP190*^*H4-1 *^background or the CP190^3^/CP190^P1 ^background which gives a few escaper adults. In both the *CP190*^*H4-1 *^and CP190^3^/CP190^P1 ^mutants, adults containing the GFP- or Myc-tagged CP190dBTB transgenes have the same *y*^*2 *^and *ct*^*6 *^phenotypes as the mutant without the transgenes (data not shown), indicating that the BTB domain is required for insulator activity.

### The BTB, but not the zinc finger or CENT domains, is essential for association of Cp190 with the Su(Hw)-Mod(mdg4)67.2 insulator complex

**Figure 3:**
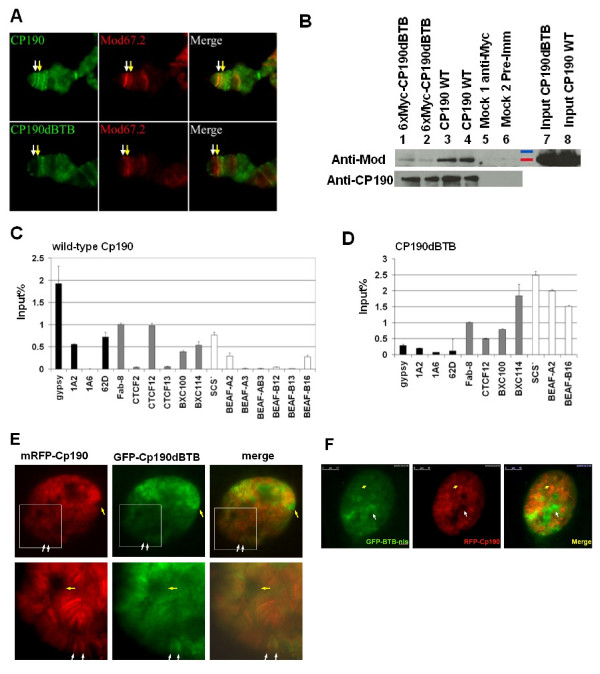
**The BTB domain is necessary but not sufficient for association with the Su(Hw) complex**. (A) Distribution of myc-CP190dBTB and Cp190 on polytene chromosomes. The polytene chromosomes of the *y*^*2 *^(upper panel) and of the *y*^*2*^*; P[Ubi63e::myc-CP190dBTB]; CP190*^*P1*^*/CP190*^*3 *^(lower panel) flies were stained with anti-CP190 (left column) and anti-Mod(mdg4)67.2 (middle column). Shown are the tips of X chromosomes with the *y *locus (white arrows) and a band nears *y *(yellow arrows). (B) Co-immunoprecipitation of Cp190 and Mod(mdg4)67.2. Immunonblots of anti-Cp190 (top panel) and anti-Mod(mdg4)67.2 (bottom panel). Proteins were immunoprecipitated with anti-Myc from *y*^*2 *^*ct*^*6*^*; P[Ubi63e::myc-CP190dBTB]/+; CP190*^*3*^*/TM6B *pupae (lanes 1 and 2), with anti-Cp190 from *y*^*2 *^*ct*^*6 *^pupae (lanes 3 and 4), and with anti-Myc (lane 5) or pre-immune (lane 6) from *y*^*2 *^*ct*^*6 *^pupae. Input controls from myc-CP190dBTB (lane 7) and from *y*^*2 *^*ct*^*6 *^(lane 8) pupae. (C-D) Anti-Cp190 ChIP of known Su(Hw), CTCF, and BEAF32 loci assayed by Real-Time PCR (percentage of input DNA, n≥3), from *y*^*2 *^*ct*^*6 *^flies (C) and from *ct*^*6*^*; P[Ubi63e::myc-CP190dBTB; CP190*^*3*^*/CP190*^*P1 *^flies (D). The 1A6 region is the negative control [12]. All results were normalized to *Fab-8*. (E) Distribution of the mRFP-CP190 (red, left column), GFP-CP190dBTB (green, middle column) in a living salivary gland cell nucleus from a 3^rd ^instar larva. An extra-chromosomal space containing GFP-CP190dBTB signals but not mRFP-CP190 signals (yellow arrows). The closer views (bottom row) are crops indicated by the white squares in the upper row. The white arrows point to two bands containing both mRFP-CP190 and GFP-CP190dBTB. (F) Distribution of GFP-CP190BTB-nls (green, left) and mRFP-CP190 (red) in the cell nucleus of a living salivary gland from a 3^rd ^instar larva. The white arrows point an extra-chromosomal space containing GFP-CP190BTB-nls but not mRFP-CP190. The yellow arrows point to two mRFP-CP190 bands on polytene chromosomes that did not have detectable GFP-CP190BTB-nls signals.

The *y *locus at the tip of the X chromosome contains a *gypsy *insertion in *y*^*2 *^flies. Proteins in the Su(Hw) insulator complex including Su(Hw), Mod(mdg4)67.2 and Cp190 can be detected at the *y *locus in *y*^*2 *^flies by immunostaining of salivary gland polytene chromosomes [11]. We used immunostaining of *y*^*2 *^polytene chromosomes to assay association between the mutated Cp190 proteins and the Su(Hw) complex. We found that both the GFP-CP190dZnF and the CP190ΔM proteins bind to the *y *locus (Figure 2B), indicating that the CENT domain and the zinc fingers are not required for association of Cp190 with the *gypsy *insulator, consistent with the genetic complementation results which show that these domains are not essential for *gypsy *chromatin insulator activity. In contrast the myc-CP190dBTB protein was no longer present at the *gypsy *site in the *y *locus (Figure 3A, white arrows), indicating that the association of the myc-CP190dBTB protein with the Su(Hw) complex at the *gypsy *insulator is weak or non-existent.

In addition, we noticed that the myc-CP190dBTB protein still associated with many sites on chromosomes although it was absent from the Su(Hw) complex at *gypsy*, suggesting that other regions in Cp190 may mediate binding to other types of chromosome-associated complexes. We compared the distribution of the GFP-CP190dBTB and the mRFP-CP190 proteins in living cells of salivary glands dissected from 3^rd ^instar larvae. The fully functional mRFP-CP190 is associated with polytene chromosomes as multiple bands in the cell nucleus, but is not detectable in extra-chromosomal spaces (Figure 3E, yellow arrows). Although significant amounts of the GFP-CP190dBTB protein were detected on polytene chromosomes and colocalized with the mRFP-CP190 (Figure 3E, bottom row), it had a more diffuse pattern and could be detected extra-chromosomally (Figure 3E, yellow arrows). This result is consistent with the immunostaining result of polytene chromosomes which shows that CP190dBTB still associates with polytene chromosomes at many sites.

The polytene staining results described above indicate that the CP190dBTB protein does not associate with the Su(Hw)-Mod(Mdg4)67.2 complex at *gypsy*, which is supported by immunoprecipitation assays. We showed previously that proteins in the Su(Hw) complex, such as Su(Hw) and Mod(mdg4)67.2, co-precipitated with Cp190 [11]. We precipitated the myc-CP190dBTB protein with anti-MYC from extracts of the *y*^*2 *^*w ct*^*6*^*;P[Ubi63e:: myc-CP190dBTB, mini-w*^*+*^*]/+; CP190*^*3*^*/TM6B, Tb *pupae (Figure 3B, lanes 1 and 2) and detected very weak signals of co-precipitated Mod(mdg4)67.2, in contrast to precipitation of wildtype Cp190 (Figure 3B, lanes 3 and 4). The anti-Myc and anti-Cp190 immunoprecipitation reactions were specific since neither Cp190 nor Mod(mdg4)67.2 were precipitated from the *y*^*2 *^*w ct*^*6 *^pupae with anti-Myc (Figure 3B, lane 5, Mock 1) or with pre-immune serum (Figure 3B, lane 6, Mock 2). The results indicate that association of the myc-CP190dBTB with the Mod(mdg4)67.2-containing complex is significantly weaker than wild-type Cp190.

### Role of BTB domain in the association of Cp190 with multiple types of Cp190-containing insulator complexes

Cp190 associates with diverse insulators including Su(Hw), CTCF and BEAF32 [14-17]. To more closely investigate the role of the BTB domain in association between Cp190 and the three types of Cp190-containing insulator complexes, we performed chromatin immunoprecipitation (ChIP) assays (Figure 3C, D, and Supplemental Table S2 in Additional file 1). We tested Su(Hw)-associated *gypsy *loci, 1A2 and 62D [12,22,23], CTCF-associated Fab-8, CTCF2, CTCF12, CTCF13, BXC100 and BXC114 loci [17,24], and BEAF32A- or BEAF32B-associated scs', BEAF-A2, BEAF-A3, BEAF-AB3, BEAF-B12, BEAF-B13 and BEAF-B16 loci [25]. We included a site in chromosome locus 1A6 as a negative control [12]. Signals from all loci were normalized to the signal of *Fab-8 *to reveal the relative strength of association of Cp190 with tested sites in comparison with the association of Cp190 with the *Fab-8 *region. The results indicate that Cp190 associates with Su(Hw) complexes at *gypsy*, 1A2 and 62D, but not with the 1A6 negative control region (Figure 3C, black bars). Cp190 also associates with CTCF sites at Fab-8, CTCF12, BXC100, BXC114, but not at CTCF2 and CTCF13 (Figure 3C, grey bars). Cp190 binds to BEAF32 sites at scs', A2, and B16, but not at A3, AB3, B12, and B13 (Figure 3C, white bars). Association with the tested regions is specific and we did not detect these sites in ChIP samples precipitated with pre-immune serum (Supplemental Table S2 in Additional file 1).

We next determined the binding of the myc-CP190dBTB at the Cp190-positive sites and the negative control 1A6 site. The signal of myc-CP190dBTB at Fab-8 is significantly higher than the 1A6 negative control region, suggesting that substantial amounts of the myc-CP190dBTB protein lacking the BTB domain still associates with the Fab-8 region (Figure 3D). The signal of Fab-8 is weaker than those of BXC114, SCS', BEAF-A2 and BEAF-B16. Since the signal of the wild-type Cp190 at Fab-8 is stronger than the signals at BXC114, SCS', BEAF-A2 and BEAF-B16, the results indicate that the BTB domain contributes partially to the association of Cp190 with Fab-8, although the domain is not critical for the association.

In contrast to the CTCF and BEAF32 sites, signals of the three tested Su(Hw) sites (*gypsy*, 1A2, and 62D) are significantly weaker than the signal at Fab-8 (Figure 3D, black bars) and are indistinguishable with the negative control region 1A6, suggesting that the BTB domain is critical for association of Cp190 with the Su(Hw) complexes at these loci, consistent with the results of co-IP experiments and the polytene chromosome staining experiments.

The CP190dBTB protein lacking the BTB domain does not associate with the Su(Hw) complex. We thus tested if the BTB domain is sufficient to associate with insulators. We generated flies carrying the *P[Ubi63e::GFP-CP190BTB-nls, w*^*+*^*] *which encodes the fusion protein containing the GFP and the BTB domain of Cp190 fused to the nuclear localization signal of the *Drosophila *Transformer protein (GFP-CP190BTB-nls) (Figure 1A and 1C). Distribution of this GFP-tagged Cp190 mutant protein in the cell nucleus is significantly different from that of the mRFP-CP190. First, the GFP-CP190BTB-nls protein localizes to extra-chromosomal spaces but the mRFP-CP190 does not (Figure 3F, white arrows). Second, the GFP-CP190BTB-nls is not present at most of the strong mRFP-CP190 bands on polytene chromosomes in the cell nucleus (Figure 3F, yellow arrows). Third, we could not detect signals of the GFP-CP190BTB-nls protein, stained by the anti-GFP antibody, on the polytene chromosomes spreads (data not shown). These results suggest that the BTB domain alone is not sufficient to associate with the Su(Hw) insulator complexes.

### The BTB domain and an Aspartic acid-rich (D-rich) region of Cp190 are sufficient for association with *gypsy*, CTCF and BEAF32 sites

**Figure 4:**
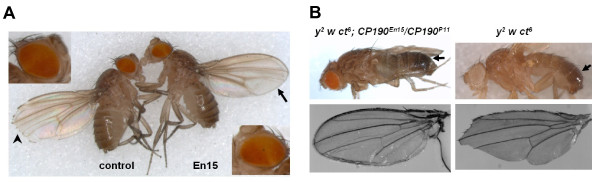
**The C-terminal E-rich domain is essential to Cp190's insulator function**. (A) Genetic assays to test functionality of the *gypsy *insulator in *CP190*^*E15*^. Shown are morphological phenotypes of the *y*^*2 *^*w omb*^*P1-D11 *^*ct*^*6*^*; mod(mdg4)*^*T6 *^*e *female (left, control) and the *y*^*2 *^*w omb*^*P1-D11 *^*ct*^*6*^*; CP190*^*En15 *^*mod(mdg4)*^*T6 *^*e/mod(mdg4)*^*T6 *^*e *female (right, En15). The En15 fly has a darker abdomen cuticle color (enhanced *y*^*2 *^phenotype) compared to the control fly. The arrowhead points to the partially suppressed *ct*^*6 *^wing shape phenotype which lacks some wing margin bristle cells. The arrow points to the fully suppressed *ct*^*6 *^wing shape (enhanced *ct*^*6 *^phenotype). The *omb*^*P1-D11 *^pigmentation pattern of the eye of the control female fly is shown on the upper left and the *omb*^*P1-D11 *^pigmentation pattern of the En15 female which has expanded white region in the equatorial part of the eye (enhanced *omb*^*P1-D11 *^phenotype) is shown on the lower right. (B) The body cuticle pigmentation (upper panel) and the wing shape (lower panel) of the *CP190*^*En15*^/CP190^P11 ^mutant (left column) and *CP190*^*+ *^flies (right column). Arrows point to different pigmentation of the abdomens.

The predicted protein of CP190^En15^, labeled as CP190dCT(En15) (Figure 1A), contains the BTB and CENT (centrosomal-association) domains, but lacks two of the three zinc fingers and the C-terminal E-rich domain. Genetic tests indicate that the CP190dCT(En15) protein cannot support insulator activity. Loss-of-function *CP190 *mutations dominantly enhance the effects of the homozygous *mod(mdg4)*^*T6 *^mutation on *gypsy*-dependent phenotypes [11]. The *CP190*^*En15 *^allele was obtained in a newly conducted genetic screen of EMS-mutagenized flies for dominant enhancers of *mod(mdg4)*^*T6*^. The *CP190*^*En15 *^mutation dominantly enhances *y*^*2*^, *omb*^*P1-D11*^, and *ct*^*6 *^all three *gypsy*-dependent phenotypes in *CP190*^*En15*^*/+*; *mod(mdg4)*^*T6 *^flies, indicating that the *gypsy *insulator function is reduced (Figure 4A). Homozygous *CP190*^*En15 *^is pupal lethal, but we found four halfway eclosed *CP190*^*En15*^/*CP190*^*P11 *^adults that survived for some 18 hours without significant locomotion after removal from the pupal case. The cuticle color of these *y*^*2 *^*w ct*^*6*^*; CP190*^*En15*^*/CP190*^*P11 *^adults was darker than the *y*^*2 *^*w ct*^*6 *^flies (Figure 4B, upper panel) and the wings had fully developed margins (Figure 4B lower panel), indicating that the *gypsy *insulator was non-functional.

**Figure 5:**
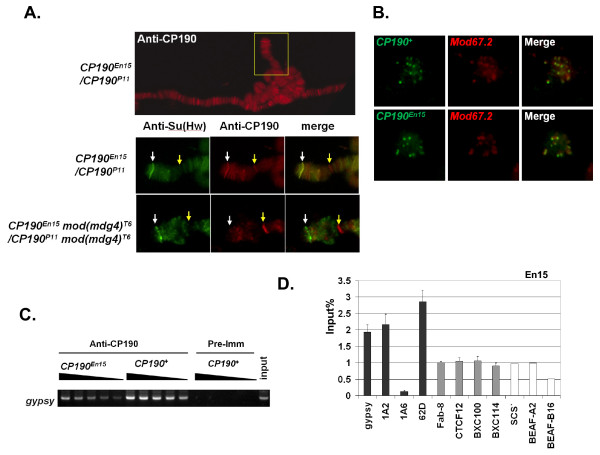
**The Cp190 fragment lacking the C-terminal E-rich domain localizes to Cp190-containing nuclear complexes**. (A) Polytene chromosomes from *y*^*2*^*; CP190*^*En15*^*/CP190*^*P11 *^(top and middle panels) and from *y*^*2*^*; CP190*^*En15 *^*mod(mdg4)*^*T6*^*/CP190*^*P11 *^*mod(mdg4)*^*T6 *^(bottom panel) 3^rd ^instar larvae were stained with anti-Su(Hw) (green, left column) and anti-Cp190 (red, middle column) antibodies. Merged images are shown on the right. The middle panel is the closer view, rotated 90 degrees counter clockwise, of the area indicated by the yellow square in the top image. White arrows point to the *y *locus at the tip of the X chromosome. Yellow arrows point to the Cp190-positive band at the constriction of the cytological location 3C. (B) Diploid cells of brain tissues from *CP190*^*+ *^flies (top) or from the *CP190*^*En15*^*/CP190*^*P11 *^(bottom) flies were stained with anti-Cp190 (green, left column) and anti-Mod(mdg4) (red, middle column) antibodies. Merged images are shown on the right. (C) The *gypsy *fragment amplified by PCR from anti-Cp190 precipitated chromatin from *y*^*2*^*; CP190*^*En15*^*/CP190*^*P11 *^pupae (left lanes, labeled *CP190*^*En15*^) and from *y*^*2*^*; CP190*^*+ *^pupae (middle lanes). The *gypsy *fragment amplified from the pre-immune serum precipitated *y*^*2 *^pupae (right lanes, labeled *CP190*^*+ *^and Pre-Imm) was the negative control and the *gypsy *fragments amplified from non-precipitated total *y*^*2 *^pupal genomic DNA (labeled as input on top) was the positive control. The black triangles indicate two-fold serial dilutions of the amount of the chromatin sample used in each PCR reaction. More volumes of the indicated template were in the PCR reactions on the left as indicated by the thicker part of each triangle. (D) The anti-Cp190 ChIP of *y*^*2 *^*ct*^*6*^*; CP190*^*En15 *^flies analyzed by Real-Time PCR. The same loci tested in Figure 3 ChIP assays were analyzed and were shown as percentage of input DNA (n ≥ 3). The results of all regions were normalized to *Fab-8*.

Although the *gypsy *insulator is non-functional in *CP190*^*En15 *^flies, the CP190dC(En15) protein is still present at *gypsy *insulators. CP190dC(En15) binds polytene chromosomes (Figure 5A, top) and colocalizes with the Su(Hw) protein at the y locus in *y*^*2 *^mutants (Figure 5A, white arrows). CP190dC(En15) also co-localizes with Su(Hw) and Mod(mdg4)67.2 proteins in diploid cells (Figure 5B). In contrast, the CP190dC(En15) protein is no longer present at the y locus of the y^2 ^polytene chromosome in the mod(mdg4) mutant (Figure 5A, bottom panel). This result supports the idea that the interaction between the BTB domains of Cp190 and Mod(mdg4)67.2 contributes to the binding of Cp190 with the Su(Hw) insulator complex. BTB domains often mediate dimers with other BTB-containing proteins, and thus we posit that the Cp190 BTB domain interacts with the Mod(mdg4)67.2 BTB domain and that Mod(mdg4)67.2 recruits Cp190 lacking the C-terminal E-rich domain.

ChIP assays with homozygous *CP190*^*En15 *^pupae indicate that CP190dC(En15) associates with all sites that bind wild-type Cp190 (Figure 5C and 5D, and Supplemental Table S4 in additional file 1), because the signals of all tested sites were significantly higher than the 1A6 negative control region. The signals at 1A2 and 62D were stronger than Fab-8, whereas in the wild-type Cp190 ChIP results the signals at 1A2 and 62D were weaker than Fab-8 (Figure 3C and 5D). The result suggests that the C-terminal E-rich domain contributes partially to the association of Cp190 with the CTCF complexes at Fab-8.

**Figure 6:**
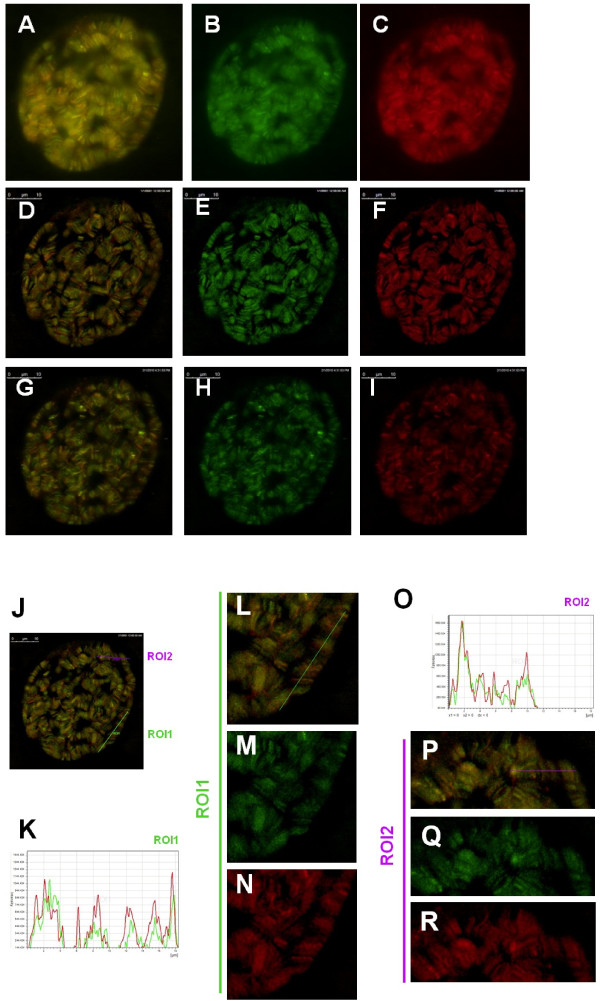
**The N-terminal Cp190 fragment containing the BTB domain and D-rich region colocalizes with the full-length Cp190 protein**. (A-I) The distribution of the GFP-CP190BTB-D (green, B, E, H, M, Q) and the mRFP-CP190 (red, C, F, I, N, R) proteins in the cell nucleus of a living salivary gland. (D-F) and (G-I) are two of the optical sections from the same cell shown in (A-C) and were analyzed by deconvolusion algorism. The same deconvolusion processed optical section as (D-F) is marked with the ROI1 (green) and ROI2 (purple) (J). (K-R) The intensity profile chart of the ROI1 (K) and the closer views of ROI1 (L-N). The ROI1 is indicated as a green line in (J) and in (L). The Intensity profile chart of the ROI2(O) and the closer views of ROI2 (P-R). The ROI2 is indicated as a purple line in (P) and in (J).

The CP190dC(En15) protein associates with all Cp190-containing insulator complexes but the GFP-CP190BTB-nls does not. We thus reasoned that another part of the Cp190 protein in addition to the BTB domain must also be essential for the association. We noticed that there is a D-Rich acidic region between the zinc fingers and the BTB domain. This D-rich region is in the CP190dC(En15) protein, but not in the GFP-CP190BTB-nls protein (Figure 1A). We generated flies carrying the *P[Ubi::GFP-CP190BTB-D, w+] *which encodes a Cp190 fragment containing both the BTB and the D-rich domain (Figure 1A). GFP-CP190BTB-D protein localizes to polytene chromosomes as distinct bands and not to extra-chromosomal spaces in living salivary glands (Figure 6). In addition, this GFP-fusion protein co-localized completely with the mRFP-CP190 on polytene chromosomes (Figure 6J-R). In diploid larval cells, e.g. brain cells and imaginal disc cells, the GFP-CP190-BTB-D protein exists as speckles and co-localizes with mRFP-CP190 (data not shown). These results indicate that this N-terminal Cp190 fragment is sufficient to associate with most of the Cp190-containing insulator complexes in living cells.

Although it associates with all Cp190 sites, GFP-CP190BTB-D, like the CP190dC(En15), is not functional in the insulator complexes and lacks essential Cp190 functions. *y*^*2 *^*w ct*^*6*^; *P[Ubi::GFP-CP190BTB-D, w+]/+; CP190*^*H4-1 *^flies have the same *y*^*2 *^body cuticle pigmentation and *ct*^*6 *^wing shape phenotypes as the *y*^*2 *^*w ct*^*6*^; *PCP190*^*H4-1 *^flies (data not shown). The GFP-CP190-BTB-D transgene also does not rescue the lethality of homozygous *CP190*^*3*^. From at least 500 F1 offspring flies of the *y*^*2 *^*w ct*^*6*^*; P[Ubi63e::GFP-CP190BTB-D, mini-w*^*+*^*]/+; CP190*^*3*^*/TM6B, Tb *parents, we obtained no *CP190*^*3 *^homozygous adults.

### The mRFP-CP190 redistributed to extra-chromosomal spaces during heat-shock whereas the CP190BTB-D fragment remained associated

**Figure 7:**
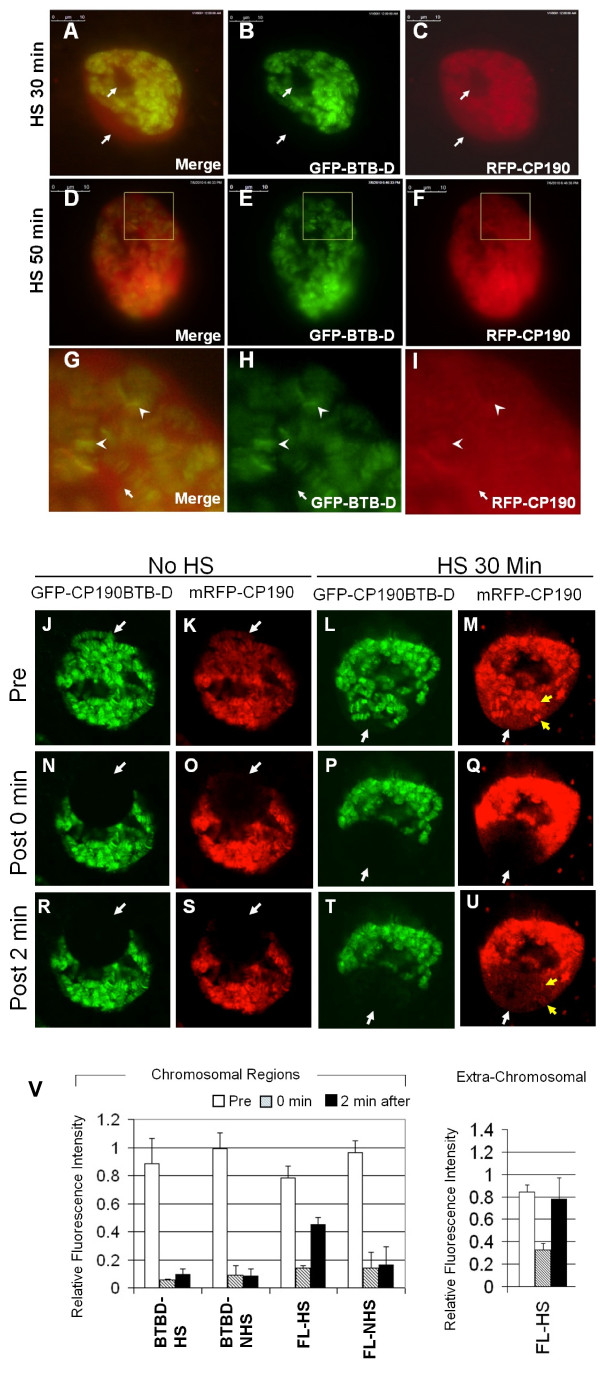
**The full sized Cp190 dissociated from polytene chromosomes during heat-shock while the CP190BTB-D fragment remained bound**. (A-I) Salivary glands dissected from 3^rd ^instar larvae expressing both mRFP-CP190 and GFP-CP190BTB-D proteins were treated with heat-shock for 30 minutes (A-C), for 50 minutes (D-I). (J-V) Salivary glands without heat-shock (J, K, N, O, R, S) or heat-shock for 30 mins (L, M, P, Q, T, U) were analyzed by the Fluorescence Recovery After Photobleach (FRAP) technique. The distribution of GFP-CP190BTB-D (green, B, E, and H) and the distribution of mRFP-CP190 (red, C, F, I) indicate that mRFP-CP190 dissociated from the chromosomes and was present in extra-chromosomal spaces (arrows). The squares in D, E, and F mark the region that is enlarged in G, H, and I. The arrow heads in G, H, and I points to two randomly sampled bands of GFP-CP190BTB-D on polytene chromosomes. (J-U) Salivary glands without the pretreatment of heat-shock (J, K, N, O, R, S) or heat-shocked for 30 minutes (L, M, P, Q, T, U) were analyzed by the FRAP technique. Images were taken before photobleaching (J, K, L, M), right after photobleaching (N, O, P, Q) and 2 minutes after photobleaching (R, S, T, U). White arrows point to areas that were photobeached. Yellow arrows point to bands reappeared after photobeaching. (V) Quantitative analysis of the fluorescence of GFP-CP190BTB-D (BTBD) and mRFP-CP190 (FL) before photobleaching (white bars), 0 minute (grey bars) and 2 minutes (black bars) after photobleaching from multiple nuclei (n > = 3) in heat-shocked treated (HS) or non-heat-shocked treated (NHS) salivary glands. The Relative Fluorescence Intensity (RFI) of chromosomal regions (left chart) and the RFI of the mRFP-CP190 in HS nuclei in extra-chromosomal regions (right chart).

The heat shock response in the *Drosophila melanogaster *has been intensively-studied. When fruit flies are stressed with heat, the transcription of most of the normal genes in cells is shut off and newly synthesized RNA species correspond to a small number of heat-induced genes [26,27]. The phenomenon of global changes in transcription is correlated with increased phosphorylation of the histone H3 Serine 10 (H3S10) at the heat-induced loci and with a sharp decrease of the global level of H3S10 phosphorylation at other loci [28]. We hypothesized that the global changes of transcription may involve changes in chromatin insulators at a global level. We thus monitored the distribution of mRFP-CP190 and GFP-CP190BTB-D proteins in cells of the salivary gland after heat-shock. We found that after 30 minutes of heat shock at 37°C, significant amounts of mRFP-CP190 localized to the extra-chromosomal space (Figure 7A-C, white arrows), although association of the protein with chromosomes was still obvious. After 50 minutes of heat shock, the mRFP-CP190 signals were mostly diffused and the protein was clearly present at extra-chromosomal spaces (Figure 7D-I, arrows). The result indicates that the heat treatment induced dissociation of the Cp190 protein from the originally bound insulator sites on chromosomes. On the other hand, we did not detect significant changes of the distribution of the GFP-CP190BTB-D protein which remained bound to polytene chromosomes as sharp bands and was not detectable in the extra-chromosomal spaces (Figure 7E and 7H, arrow heads).

To determine if Cp190 tightly associates with chromosome without heat-shock treatment, we analyzed the exchange rates of GFP-CP190BTB-D and mRFP-CP190mRFP using the Fluorescence Recovery After Photobleaching (FRAP) technique. We did not detect significant recovery of both GFP-CP190BTB-D and mRFP-CP190 signals in the bleached area two minutes after photobleaching, indicating that no significant exchanges of the two Cp190 proteins within two minutes on chromosomes (Figure 7J, K, N, O, R, S, white arrows, and Figure 7V left chart, BTBD-NHS and FL-NHS).

In the cells heat-shocked for 30 minutes, we detected signals of extra-chromosomal mRFP-CP190 (Figure 7M). The signals were significantly weaker in the bleached area right after photobleaching (Figure 7Q, and V, right chart), indicating that the extra-chromosomal signals were not background and were real signals representing the mRFP-CP190 molecules which were not associated with chromosomes. The result is consistent with the conclusion above that Cp190 may dissociate from chromosomes in response to a heat-shock treatment.

In contrast with the non-heat-shocked cells, we detected significant recovery of mRFP-CP190 signals in the bleached area within 2 minutes (Figure 7U and 7V, FL-HS), indicating that a fraction of the mRFP-CP190 rapidly moved into the bleached area. The result indicates that the heat-shocked cells contained a fraction of fast-moving mRFP-CP190 which was not present in cells before the heat treatment.

The redistributed mRFP-CP190 molecules in the bleached area were either in extra-chromosomal space where Cp190 may move more freely or were associated with chromosomes during the recovering period. It is noticeable that the distribution pattern of the recovered signals in the bleached area was different from the pattern before photobleaching (Figure 7M and 7U). In most of the bleached area, the signals that reappeared lacked distinct bands. These signals might represent mRFP-CP190 in extra-chromosomal space. However, a few bands reappeared at locations overlapping with bands that existed before photobleaching (Figure 7M and 7U, yellow arrows), implying that the mRFP-CP190 may be exchanged at a higher rate at these locations on chromosomes. All evidence from the heat-shock treatment indicates that a mechanism exists for regulating the association/dissociation of Cp190 with chromosomes. In contrast with the mRFP-CP190, the GFP-CP190BTB-D protein in the heat-shocked gland cells remained bound to chromosomes tightly. We didn't detect significant recovery of the GFP-CP190BTB-D signal in the bleach area 2 minutes after photobleaching. This result is similar to the non-heat-shocked cells (Figure 7R, T, and 7V left chart BTBD-HS), suggesting that the CP190BTB-D lacking the C-terminal E-rich domain of Cp190 is incapable of responding to the heat-shock treatment and thus remained associating with chromosomes.

## Discussion

### The BTB domain of Cp190 has multiple essential roles for fly development in addition to the association of Cp190 with the Su(Hw) complex

Multiple lines of evidence indicate that the BTB domain is required for association of Cp190 with the Su(Hw) insulator complex: (1) the CP190dBTB protein which lacks the BTB domain does not associate with the *gypsy *insulator sequence in ChIP assays and does not localize to the *gypsy *site on polytene chromosomes; (2) proteins in the Su(Hw) complex are not co-precipitated with myc-CP190dBTB, but are co-precipitated with wild-type Cp190. Lack of association between the CP190dBTB protein and the Su(Hw) complex at the *gypsy *insulators in a *CP190 *mutant may result in defective functionality of the insulator which is also supported by the genetic complementation result that expression of the protein does not rescue the defective *gypsy *insulator activity in homozygous *CP190 *mutants. It is likely that the BTB domain interacts with the BTB domain of Mod(Mdg4)67.2 because Mod(mdg4)67.2 lacking the BTB domain fails to interact with Cp190 in two-hybrid assays and is not functional in vivo [29].

In addition to the critical role in the association of Cp190 with the Su(Hw) complex, the BTB domain of Cp190 must have other essential roles for viability of flies. This is because the homozygous *su(Hw) *null is female sterile, however *CP190*^*3 *^flies expressing the GFP-CP190dBTB or myc-CP190dBTB proteins are still inviable, indicating that the CP190dBTB proteins are unable to support at least one function for viability in other Cp190-containing complexes. Both the polytene staining results and ChIP assays from myc-CP190dBTB indicate that the BTB domain is not essential for association with the CTCF or BEAF32 complexes, but quantitatively contributes to the association with these complexes. Thus either the CTCF or BEAF32 complexes containing the myc-CP190dBTB are defective in function or the BTB domain is involved in an activity essential for fly survival but unrelated to the three types of insulators.

### The E-rich domain contributes quantitatively to the association of Cp190 with all three types of insulator complexes and is essential for Cp190's functions

The C-terminal E-rich region is not necessary for the association of Cp190 with all three types of insulator complexes, because the CP190dCT(En15) fragment that lacks the whole E-rich region localizes to all the tested Cp190 wild-type containing Su(Hw), CTCF and BEAF sites in ChIP assays. This conclusion is well supported by the complete co-localization of the GFP-CP190BTB-D fragment with the mRFP-CP190 full-length protein on polytene chromosomes in the living salivary gland cell nucleus. The E-rich domain however may still contribute to the association of Cp190 with the Su(Hw) complex since the Cp190 wild-type protein still associates with the Su(Hw) complex in the *mod(mdg4)*^*u1 *^mutant [11], but the CP190dC(En15) fragment lacking the E-rich region does not. The interaction between the E-rich region and the Su(Hw) protein may stabilize Cp190 in the Su(Hw) insulator complex, although the interaction is not essential for association. More importantly, the E-rich domain is required for the essential function of Cp190 because the homozygous CP190^En15 ^fly is lethal and the *P[Ubi::GFP-CP190BTB-D, w+] *transgene does not rescue the lethality of the homozygous *CP190*^*3 *^mutant. It is likely that the E-rich domain is required by all the Cp190-containing insulator complexes.

### The dissociation of Cp190 with chromosomes is a regulated process and requires the function of the E-rich domain

ChIP-chip results from several groups published recently showed that not all Su(Hw) complexes, CTCF complexes or BEAF32 complexes contain Cp190 [16,17]. We also found that some tested chromatic regions containing CTCF complexes or BEAF32 complexes which were not associated with significant amounts of Cp190. This phenomenon argues that the recruitment of Cp190 to each individual insulator site may be regulated. This view is supported by the dynamic distribution of Cp190 during heat-shock. Significant amounts of mRFP-CP190 may dissociate from bound sites and localize to the extra-chromosomal space, implying that a mechanism exists for regulating the association/dissociation of Cp190 with chromosomes.

Cp190 binds tightly to chromosomes when flies were cultured in normal temperature. We didn't detect significant exchange of either the full-size Cp190 protein or the CP190BTB-D fragment on chromosomes. In cells treated with heat-shock, the full-size Cp190 protein dissociated from chromosomes and redistributed into the extra-chromosomal space. This indicates that dissociation of Cp190 is a regulated process. In the same heat-shocked cells, CP190BTB-D which lacks the C-terminal part of Cp190 was still tightly bound to chromosomes while the full-size Cp190 dissociated. This phenomenon strongly suggests that the C-terminal part of Cp190 must be essential for the dissociation. A possible mechanism for this phenomenon is that modifications to the C-terminal part of Cp190, for example phosphorylation, would weaken the interaction between Cp190 and other proteins in insulator complexes. Genetic evidence indicates that insulator complexes without Cp190 are not functional. Dissociation of Cp190 therefore may down-regulate activities of insulators thus affecting the expression of local genes. Further characterization of the interactions will be necessary to understand the molecular mechanism through which Cp190 is recruited differently to the insulator complexes at different genetic locations. However since relatively less information about the composition of the CTCF and the BEAF32 complexes is known, more detailed analysis of the molecular interactions will require identification of more components in the two types of chromatin insulator complexes.

## Conclusions

We have determined sub-regions of the Cp190 protein required for fly survival, for association with Cp190-containing insulators and for the *gypsy *insulator activity. The N-terminal CP190BTB-D fragment of Cp190 containing the BTB domain and the D-rich acidic region is sufficient for association with chromosomes. The fragment however is insufficient for insulator activity and for fly survival during development. The middle portion of the Cp190 protein, including the CENT domain which mediates centrosomal localization and the zinc finger domain, is dispensable for critical insulator functions. The C-terminal E-rich acidic region strengthens association of Cp190 with most insulator sites and is essential for Cp190's insulator function.

We have shown evidence that dissociation of Cp190 from its bound sites on chromosomes is a regulated process. Cp190 dissociated from chromosomes when cells were treated with heat-shock. In contrast, the CP190BTB-D lacking the E-rich domain did not dissociate from chromosomes during heat-shock, indicating that the E-rich region is required for this dissociation process. Previous findings have demonstrated that the function of chromatin insulators requires association of Cp190 with insulator sites. Our results provide a mechanism through which the activities of Cp190-containing chromatin insulators may be regulated.

## Methods

### Antibodies

Rabbit and rat anti-Cp190 antibodies were reported previously [11]. A rat anti-CP190BTB-D antibody was used for the immunoblot in Figure 1B. The antibody was generated by immunizing rats (Pocono Rabbit Farm and Laboratory Inc.) with the 6X-His-CP190BTB-D fusion protein purified from the *BL21 *E. coli transformed with pET15B.CP190BamHI in which a BamHI digested CP190 cDNA was inserted in frame into pET15B vector. One of the rabbit anti-Cp190 antibodies was successfully used in immunoprecipitation experiments [11] and in ChIP assays [17]. The rabbit anti-Cp190 antibody was used in the ChIP assays, immunofluorescence stainings of polytene chromosomes, and immunoprecipitation experiments in this study. The rat anti-Mod(mdg4)67.2 polyclonal antibody was reported earlier [30]. The rat anti-actin antibody in immunoblots was purchased from Abcam Co. (ab50591-100). The rabbit-anti-GFP antiserum was raised by immunizing rabbits with purified bacteria-expressed His-GFP protein (Pocono Rabbit Farm and Laboratory Inc.).

### The *CP190 *mutants

P-elements containing *CP190 *truncations were generated by inserting the full-length or truncated CP190 cDNA fragments into pENTR/D-Topo (Invitrogen) which were subsequently recombined with pUGW or pURW destination vectors [20]. All P-elements obtained were introduced into flies with the traditional germ line transformation procedures and were crossed into CP190 deficient background by classical genetic manipulation. Flies were cultured in 23°C or 26°C environmental chambers.

To generate the P-element encoding the GFP-CP190dBTB, we performed PCR using the full-length *CP190 *cDNA (LD02352, Research Genetics) as the template and the 5'-caccgagaacgttaatcgccag-3' and 5'-tagctcctccttcgccgc-3' as the primers. The amplified CP190dBTB fragment was inserted into pENTR/D-Topo vector (Invitrogen) to obtain the entry clone pENTR.CP190dBTB. The pENTR.CP190dBTB was recombined with destination vectors pUMW or pUGW vectors [20] using Clonase II (Invitrogen) to become pUMW.CP190dBTB for generating flies carrying *P[Ubi63e::myc-CP190dBTB, w*^*+*^*] *or pUGW.CP190dBTB for generating flies carrying *P[Ubi63e::GFP-CP190dBTB, w*^*+*^*]*. To generate the deletion of zinc fingers in the Cp190 protein, the CP190 full-length cDNA in the pBluescript SK^- ^vector was mutagenized with the Quickchange XL Mutagenesis Kit (Stratagene) using 5'-gcacaaggagacaattgatgagcaggctttggaggatggc-3' and 5'-gccatcctccaaagcctgctcatcaattgtctccttgtgc-3' primers. The obtained clone (pSK-.CP190dZnF) with anticipated deletion was confirmed by sequencing. To create the entry clone pENTR.CP190dZnF, the CP190dZnF fragment in pSK-.CP190dZnF was amplified using 5'-caccagccagagcaagcgaaac-3' and 5'-tagctcctccttcgccgc-3' primers. The resulting fragment was inserted into the pENTR/D-Topo vector (Invitrogen) to generate the entry clone pENTR.CP190dZnF and the insert was subsequently recombined into pUGW [20] using Clonase II to obtain the pUGW.CP190dZnF for generating flies carrying *P[Ubi63e::GFP-CP190dZnF, w*^*+*^*]*. For flies expressing GFP-CP190BTB-nls fusion protein we performed fusion-PCR to fuse the *CP190 *cDNA fragment amplified by 5'-caccagccagagcaagcgaaac-3' and 5'-tctgtgcctgctcttggtgcgacggtgcgc-3' primers and the cDNA fragment encoding the nuclear localization sequence (NLS) of the *Drosophila melanogaster *Transformer protein amplified by 5'- gcgcaccgtcgcaccaagagcaggcacaga and 5'-gcgtcttcgttcactgct-3'. The resulting fragment was inserted into the pENTR/D-Topo to obtain the entry clone pENTR.CP190BTB-nls which was subsequently recombined with the destination vector pUGW using Clonase II to obtain the pUGW.CP190BTB-nls which was injected into flies for generating flies carrying the *P[Ubi63e::CP190BTB-nls, w*^*+*^*]*. For flies expressing the GFP-CP190BTB-D fusion protein, the *CP190 *cDNA fragment amplified by 5'-caccagccagagcaagcgaaac-3' and 5'-cgccgggggttttactgtcgctgg-3' was inserted into the pENTR/D-Topo to obtain the entry clone pENTR.CP190BTB-D which was subsequently recombined with the destination vector pUGW using Clonase II to obtain the pUGW.CP190BTB-D which was injected into flies to generate flies carrying *P[Ubi63e::GFP-CP190BTB-D, w*^*+*^*]*. The fly stocks carrying the CP190ΔM and the CP190^3 ^were obtained from Dr. J. W. Raff [19]. All the transgenic lines evaluated are on the second chromosome, except the *P[Ubi63e::mRFP-CP190, w*^*+*^*] *which were all inserted on the 3^rd ^chromosome. We recombined two independent 3^rd ^chromosome *P[Ubi63e::mRFP-CP190, w*^*+*^] transgenic insertions onto the chromosome containing the *CP190*^*3 *^mutation. Both transgenic lines express similar amounts of the encoded mRFP-CP190 fusion protein and behaved the same in the genetic complementation assays. The *CP190*^*3 *^mutation on the recombined chromosomes was confirmed by sequencing reactions using endogenous *CP190 *specific primers (data not shown) and is evident by lacking of the wild-type Cp190 protein in the protein lysates prepared from the *y*^*2 *^*w ct*^*6*^*; P[Ubi63e::mRFP-CP190, w*^*+*^*] CP190*^*3*^*/CP190*^*3 *^larvae (Figure 1B).

### Genetics and phenotypic analysis

Flies were cultured in 23°C or 26°C environmental chambers. Phenotypes of adult flies and wings were examine by the Leica S8 stereoscope and were imaged by the Leica FX280 digital camera. To obtain larger focal depth of the fly or wing images, multiple images of consecutive focal planes may be combined (Helicon focus). All images were processed with the preset condition of the software.

For the genetic complementation analysis of P[*Ubi63e::mRFP-CP190, w*^*+*^], from the genetic cross of the *y*^*2 *^*w ct*^*6*^*; P[Ubi63e::mRFP-CP190, w*^*+*^*] CP190*^*3*^*/TM6B, Tb *and *y*^*2 *^*w ct*^*6*^*; CP190*^*3*^*/TM6B, Tb *parents, we evaluated 52 adult offspring adult flies and observed 16 *Tb*^*+ *^homozygous *CP190*^*3 *^adults. The ratio (16/52) is close to the expected Mendelian ratio (1/3) if the transgene rescues. In contrast, from the control cross containing *y*^*2 *^*w ct*^*6*^*; CP190*^*3*^*/TM6B, Tb *parents, we evaluated at least 500 offspring flies and we could not find a single homozygous *CP190*^*3 *^adult.

For the genetic complementation analysis of P[*Ubi63e::GFP*-CP190dZnF, mini-w^*+*^], from the genetic cross of *y*^*2 *^*w ct*^*6*^*; P[Ubi63e::GFP-CP190dZnF, mini-w*^*+*^*]/+; CP190*^*3*^*/TM6B, Tb *parents, we evaluated 112 of the offspring flies and obtained 13 homozygous *CP190*^*3 *^adults, which is close to the expected Mendelian ratio if the transgene rescues (1/6). All 13 *CP190*^*3 *^homozygous adults were *w*^*+*^, indicating that they contain the *GFP-CP190dZnF *transgene (*y*^*2 *^*w ct*^*6*^*; P[Ubi63e::GFP-CP190dZnF, mini-w*^*+*^*]/+; CP190*^*3*^).

For the genetic complementation analysis of P[*Ubi63e::myc-CP190dBTB, mini-w*^*+*^] and P[*Ubi63e::GFP-CP190dBTB, mini-w*^*+*^], three transgenic P[*Ubi63e::myc-CP190dBTB, mini-w*^*+*^] lines and two transgenic P[*Ubi63e::GFP-CP190dBTB, mini-w*^*+*^] lines on the second chromosome were introduced into the *CP190*^*3*^*/TM6B, Tb *genetic background. We evaluated at least 500 progeny from the P[*Ubi63e::myc-CP190dBTB, mini-w*^*+*^]/+; *CP190*^*3*^*/TM6B, Tb *parents or the P[*Ubi63e::GFP-CP190dBTB, mini-w*^*+*^]/+; *CP190*^*3*^*/TM6B, Tb *parents of each transgenic line. We observed at least 100 homozygous *CP190*^*3 *^larvae and pupae in each line but could not find homozygous *CP190*^*3 *^adults, indicating that P[*Ubi63e::myc-CP190dBTB, mini-w*^*+*^] and P[*Ubi63e::GFP-CP190dBTB, mini-w*^*+*^] transgenes do not rescue lethality of the homozygous *CP190*^*3 *^mutation.

### Chromatin Immunoprecipitation

ChIP was performed from pupae (0.1 g) by dounce homogenization in 1 ml of ice-cold PBSMT (2.5 mM MgCl_2_, 3 mM KCl, and 0.3% Triton X-100 in PBS) plus protease inhibitors (Complete protease inhibitor tablet cocktail, Roche). Homogenized cells were cross-linked by 1% formaldehyde solution and were sonicated to obtain 200-1000 bps DNA fragments. ChIP was performed using the rabbit anti-Cp190 [11,17], mouse anti-MYC 500 μl (9E, Hybridoma Bank at the University of Iowa), or preimmune serum. For regular PCR analysis, DNA was serial diluted and amplified with *gypsy*-specific primers 5'-GCGCGCGAATTCGTGTGCGTTGAATTTATTCGCAAA-3' 5'-GGTATGCAATATAATAATCTTTTATTG-3' and the Fab-8 specific primers 5'- GGCACAATCAAGTTAATGTTGG-3' and 5'- GCAAGCGAAGAGTTCCATTC-3'. For Real-Time PCR analysis, the DNA samples were mixed with primers (see Supplement Table S1 in additional file 1) and Fast SYBR Green master mix (Applied Biosystems). The PCR reactions were performed in a Fast 7500 Real-Time PCR system (Applied Biosystems) using a standard program with 60°C annealing temperature and 45 seconds of elongation time.

### Microscopy

Polytene chromosome spreads were prepared as described previously [11]. For live salivary gland cell imaging, freshly dissected glands were cultured in the serum-free insect medium (Invitrogen) and were examined immediately. For the heat-shock experiments, 3^rd ^instar larvae containing the transgenes were heated in a 37°C water bath. Salivary glands from heat-treated larvae were dissected in the pre-warmed serum-free insect medium and viewed immediately under microscope. All fluorescent images were taken by the Leica DM5500 scope and the Leica FX350 camera. The deconvolusion analysis was performed with the AF6000 software (Leica) using the standard preset condition. The analysis of FRAP was performed with a confocal microscope (Olympus) and processed by the FluoView software (Olympus). For FRAP experiments, untreated or heat-shock treated (37°C water bath for the desired time) third instar larvae were dissected in a cold serum-free insect medium (Invitrogen). The dissected salivary glands were transferred into a culture chamber and were investigated immediately under the confocal microscope. For quantitative analysis of fluorescence of GFP-CP190BTB-D and mRFP-CP190 before and after photobleaching, the "Relative Fluorescence Intensities" of randomly sampled chromosomal regions or extra-chromosomal space were determined. The "Relative Fluorescence Intensity" (F_r_) was calculated as the average fluorescence of three randomly sampled spots in the bleached area (F_b_) divided by the average of three randomly sampled chromosomal reference spots in the non-bleached area (F_nb_). F_r _= F_b_/F_nb_. The F_b _and F_nb _were calculated from the same bleached spots and non-bleached reference spots in a nucleus before photobleaching, and 0 minute and 2 minutes after photobleaching.

### Characterization of the dominant enhancer phenotypes of *CP190*^*En15*^

The *CP190*^*En15 *^mutation dominantly enhances the effects of the *mod(mdg4)*^*T6 *^mutation on the *y*^*2*^*, omb*^*P1-D11*^, and *ct*^*6 *^all three *gypsy*-dependent phenotypes (Figure 4A). The altered phenotypes consistently indicate that the *gypsy *insulator has reduced functionality in the heterozygous *CP190*^*En15 *^flies: (1) wings of the *y*^*2 *^*w omb*^*P1-D11 *^*ct*^*6*^*; CP190*^*En15 *^*mod(mdg4)*^*T6*^*/mod(mdg4)*^*T6 *^flies have a wild-type shape and margins, suggesting a complete loss-of-function of the *gypsy *insulator at the *ct*^*6 *^locus (Figure 4A, the fly on the right, arrow), whereas the wings of the *y*^*2 *^*w omb*^*P1-D11 *^*ct*^*6*^*; mod(mdg4)*^*T6*^*/mod(mdg4)*^*T6 *^flies have underdeveloped margins, indicating that the *gypsy *insulator at the *ct*^*6 *^locus in the fly strain is weak but is still functional (Figure 4A, the fly on the left, arrowhead); (2) *y*^*2 *^*w omb*^*P1-D11 *^*ct*^*6*^*; CP190*^*En15 *^*mod(mdg4)*^*T6*^*/mod(mdg4)*^*T6 *^flies have more darkly pigmented body cuticle and wings than *y*^*2 *^*w omb*^*P1-D11 *^*ct*^*6*^*; mod(mdg4)*^*T6*^*/mod(mdg4)*^*T6 *^flies, indicating a weaker *gypsy *insulator activity in the *CP190*^*En15 *^*mod(mdg4)*^*T6*^*/mod(mdg4)*^*T6 *^flies; (3) in *y*^*2 *^*w omb*^*P1-D11 *^*ct*^*6*^*; CP190*^*En15 *^*mod(mdg4)*^*T6*^*/mod(mdg4)*^*T6 *^flies, the eyes have a wider un-pigmented region in the equatorial part of the eye comparing to that of the *y*^*2 *^*w omb*^*P1-D11 *^*ct*^*6*^*; mod(mdg4)*^*T6*^*/mod(mdg4)*^*T6 *^flies (Figure 4A, lower right and upper left closer views). The *omb*^*P1-D11 *^marker is a *gypsy*-dependent pigmentation pattern in the eyes [31]. The *w omb*^*P1-D11 *^female flies have evenly pigmented eyes due to a P[*lacW*] and a *gypsy *inserted in the *optomoter blind *(*omb*) locus. The partially degraded *gypsy *insulator function in the *y*^*2 *^*w omb*^*P1-D11 *^*ct*^*6*^*; mod(mdg4)*^*T6*^*/mod(mdg4)*^*T6 *^female flies results in a slightly un-pigmented area in the equatorial part of the eyes (Figure 4A, top left closer view) [11]. The phenomenon of a wider un-pigmented area in the eyes of the *y*^*2 *^*w omb*^*P1-D11 *^*ct*^*6*^*; CP190*^*En15 *^*mod(mdg4)*^*T6*^*/mod(mdg4)*^*T6 *^female flies than the *y*^*2 *^*w omb*^*P1-D11 *^*ct*^*6*^*; mod(mdg4)*^*T6*^*/mod(mdg4)*^*T6 *^female flies suggests that the insulator function in the *CP190*^*En15 *^*mod(mdg4)*^*T6*^*/mod(mdg4)*^*T6 *^flies is even weaker than in the *mod(mdg4)*^*T6*^*/mod(mdg4)*^*T6 *^flies.

## Authors' contributions

DO generated four of the constructs for tagged Cp190 mutant proteins, including GFP-CP190BTB, myc-CP190dBTB, GFP-CP190dBTB, and CP190dZnF; performed the original Western blot of the Figure 1B; determined the localization of CP190dZnF, CP190dC(En15) on polytene chromosomes with immunofluorescence staining in Figure 2B, 3A, and 5A; determined the localization of CP190dC(En15) protein in diploid cells in Figure 5B; performed immunoprecipitation experiment and revealed that CP190dBTB does not association with the Su(Hw)-Mod(mdg4)67.2 complex; precipitated the chromatin associated with Cp190 in *CP190*^*+*^, *CP190dBTB*, and *CP190*^*En15 *^flies, and performed the initial analysis of the association of the Cp190 with the *gypsy *insulator complexes in these flies. BS performed the Real-Time PCR analysis of all the tested sites with the ChIP samples; reproduced the Western blot result of Figure 1B; performed the Western blots of Figure 1C; characterized the localization of mRFP-CP190, GFP-CP190dBTB, and GFP-CP190BTB in Figure 3E and 3F; determined the localization of mRFP-CP190 and GFP-CP190BTB-D proteins in heat-shocked cells; performed photo-bleaching experiments to determined diffusion rates of mRFP-CP190 and GFP-CP190BTB-D proteins in the cell nucleus. HS generated the GFP-CP190BTB-D expression flies; characterized the localization of GFP-CP190BTB-D in polytene cells. OA determined the localization of CP190ΔM protein at the *y *locus on polytene chromosomes. CP generated the *CP190*^*En15 *^mutant and characterized the *gypsy*-related phenotypes of this mutant; supervised the progress of the project. All authors read and approved the final manuscript.

## Supplementary Material

Additional file 1 **Real-Time PCR analysis of ChIP assays**. Primers for the Real-Time PCR analysis of ChIP assays (Table S1). Raw data for the *y*^*2 *^*ct*^*6 *^anti-Cp190 ChIP (Table S2); for the myc-CP190dBTB anti-Cp190 ChIP (Table S3); for the CP190dC(En15) anti-Cp190 ChIP (S4). Click here for file

## References

[B1] Gyurkovics H Gausz J Kummer J Karch F A new homeotic mutation in the *Drosophila *bithorax complex removes a boundary separating two domains of regulation Embo J 1990 9 8 2579 2585 1973385 10.1002/j.1460-2075.1990.tb07439.x PMC552290

[B2] Geyer PK Corces VG DNA position-specific repression of transcription by a *Drosophila *zinc finger protein Genes Dev 1992 6 10 1865 1873 10.1101/gad.6.10.1865 1327958

[B3] Jack J Dorsett D Delotto Y Liu S Expression of the cut locus in the *Drosophila *wing margin is required for cell type specification and is regulated by a distant enhancer Development 1991 113 3 735 747 1821846 10.1242/dev.113.3.735

[B4] Spana C Harrison DA Corces VG The *Drosophila melanogaster *suppressor of Hairy-wing protein binds to specific sequences of the *gypsy *retrotransposon Genes Dev 1988 2 11 1414 1423 10.1101/gad.2.11.1414 2850261

[B5] Dorsett D Potentiation of a polyadenylylation site by a downstream protein-DNA interaction Proc Natl Acad Sci USA 1990 87 11 4373 4377 10.1073/pnas.87.11.4373 2161539 PMC54112

[B6] Kim J Shen B Rosen C Dorsett D The DNA-binding and enhancer-blocking domains of the *Drosophila *suppressor of Hairy-wing protein Mol Cell Biol 1996 16 7 3381 3392 8668153 10.1128/mcb.16.7.3381 PMC231332

[B7] Ghosh D Gerasimova TI Corces VG Interactions between the Su(Hw) and Mod(mdg4) proteins required for *gypsy *insulator function The EMBO journal 2001 20 10 2518 2527 10.1093/emboj/20.10.2518 11350941 PMC125459

[B8] Capelson M Corces VG SUMO conjugation attenuates the activity of the *gypsy *chromatin insulator Embo J 2006 16628226 10.1038/sj.emboj.7601068 PMC1456934

[B9] Gerasimova TI Corces VG Polycomb and trithorax group proteins mediate the function of a chromatin insulator Cell 1998 92 4 511 521 10.1016/S0092-8674(00)80944-7 9491892

[B10] Lei EP Corces VG RNA interference machinery influences the nuclear organization of a chromatin insulator Nat Genet 2006 38 8 936 941 10.1038/ng1850 16862159

[B11] Pai CY Lei EP Ghosh D Corces VG The centrosomal protein CP190 is a component of the *gypsy *chromatin insulator Mol Cell 2004 16 5 737 748 10.1016/j.molcel.2004.11.004 15574329

[B12] Kurshakova M Maksimenko O Golovnin A Pulina M Georgieva S Georgiev P Krasnov A Evolutionarily conserved E(y)2/Sus1 protein is essential for the barrier activity of Su(Hw)-dependent insulators in *Drosophila* Mol Cell 2007 27 2 332 338 10.1016/j.molcel.2007.05.035 17643381

[B13] Gause M Morcillo P Dorsett D Insulation of enhancer-promoter communication by a *gypsy *transposon insert in the *Drosophila **cut *gene: cooperation between suppressor of hairy-wing and modifier of mdg4 proteins Molecular and cellular biology 2001 21 14 4807 4817 10.1128/MCB.21.14.4807-4817.2001 11416154 PMC87172

[B14] Mohan M Bartkuhn M Herold M Philippen A Heinl N Bardenhagen I Leers J White RA Renkawitz-Pohl R Saumweber H The *Drosophila *insulator proteins CTCF and CP190 link enhancer blocking to body patterning Embo J 2007 26 19 4203 4214 10.1038/sj.emboj.7601851 17805343 PMC2230845

[B15] Gerasimova TI Lei EP Bushey AM Corces VG Coordinated control of dCTCF and *gypsy *chromatin insulators in Drosophila Mol Cell 2007 28 5 761 772 10.1016/j.molcel.2007.09.024 18082602 PMC2579779

[B16] Negre N Brown CD Shah PK Kheradpour P Morrison CA Henikoff JG Feng X Ahmad K Russell S White RA A comprehensive map of insulator elements for the Drosophila genome PLoS genetics 2010 6 1 e1000814 10.1371/journal.pgen.1000814 20084099 PMC2797089

[B17] Bushey AM Ramos E Corces VG Three subclasses of a Drosophila insulator show distinct and cell type-specific genomic distributions Genes Dev 2009 23 11 1338 1350 10.1101/gad.1798209 19443682 PMC2701583

[B18] Oegema K Whitfield WG Alberts B The cell cycle-dependent localization of the CP190 centrosomal protein is determined by the coordinate action of two separable domains J Cell Biol 1995 131 5 1261 1273 10.1083/jcb.131.5.1261 8522588 PMC2120638

[B19] Butcher RD Chodagam S Basto R Wakefield JG Henderson DS Raff JW Whitfield WG The Drosophila centrosome-associated protein CP190 is essential for viability but not for cell division J Cell Sci 2004 117 Pt 7 1191 1199 10.1242/jcs.00979 14996941

[B20] Akbari OS Oliver D Eyer K Pai CY An Entry/Gateway cloning system for general expression of genes with molecular tags in Drosophila melanogaster BMC Cell Biol 2009 10 1 8 10.1186/1471-2121-10-8 19178707 PMC2654426

[B21] Chodagam S Royou A Whitfield W Karess R Raff JW The centrosomal protein CP190 regulates myosin function during early Drosophila development Curr Biol 2005 15 14 1308 1313 10.1016/j.cub.2005.06.024 16051175

[B22] Golovnin A Birukova I Romanova O Silicheva M Parshikov A Savitskaya E Pirrotta V Georgiev P An endogenous Su(Hw) insulator separates the yellow gene from the Achaete-scute gene complex in Drosophila Development 2003 130 14 3249 3258 10.1242/dev.00543 12783795

[B23] Parnell TJ Viering MM Skjesol A Helou C Kuhn EJ Geyer PK An endogenous suppressor of hairy-wing insulator separates regulatory domains in Drosophila Proc Natl Acad Sci USA 2003 100 23 13436 13441 10.1073/pnas.2333111100 14597701 PMC263832

[B24] Holohan EE Kwong C Adryan B Bartkuhn M Herold M Renkawitz R Russell S White R CTCF genomic binding sites in Drosophila and the organisation of the bithorax complex PLoS Genet 2007 3 7 e112 10.1371/journal.pgen.0030112 17616980 PMC1904468

[B25] Jiang N Emberly E Cuvier O Hart CM Genome-wide mapping of boundary element-associated factor (BEAF) binding sites in Drosophila melanogaster links BEAF to transcription Mol Cell Biol 2009 29 13 3556 3568 10.1128/MCB.01748-08 19380483 PMC2698748

[B26] Spradling A Penman S Pardue ML Analysis of drosophila mRNA by in situ hybridization: sequences transcribed in normal and heat shocked cultured cells Cell 1975 4 4 395 404 10.1016/0092-8674(75)90160-9 1122559

[B27] McKenzie SL Henikoff S Meselson M Localization of RNA from heat-induced polysomes at puff sites in Drosophila melanogaster Proceedings of the National Academy of Sciences of the United States of America 1975 72 3 1117 1121 10.1073/pnas.72.3.1117 805422 PMC432477

[B28] Nowak SJ Corces VG Phosphorylation of histone H3 correlates with transcriptionally active loci Genes Dev 2000 14 23 3003 3013 10.1101/gad.848800 11114889 PMC317109

[B29] Golovnin A Mazur A Kopantseva M Kurshakova M Gulak PV Gilmore B Whitfield WG Geyer P Pirrotta V Georgiev P Integrity of the Mod(mdg4)-67.2 BTB domain is critical to insulator function in Drosophila melanogaster Mol Cell Biol 2007 27 3 963 974 10.1128/MCB.00795-06 17101769 PMC1800699

[B30] Mongelard F Labrador M Baxter EM Gerasimova TI Corces VG Trans-splicing as a novel mechanism to explain interallelic complementation in Drosophila Genetics 2002 160 4 1481 1487 11973303 10.1093/genetics/160.4.1481 PMC1462063

[B31] Tsai SF Jang CC Prikhod'ko GG Bessarab DA Tang CY Pflugfelder GO Sun YH *Gypsy *retrotransposon as a tool for the in vivo analysis of the regulatory region of the optomotor-blind gene in Drosophila Proc Natl Acad Sci USA 1997 94 8 3837 3841 10.1073/pnas.94.8.3837 9108065 PMC20528

