# Supplementary material for: The chromosomal association/dissociation of the chromatin insulator protein Cp190 of Drosophila melanogaster is mediated by the BTB/POZ domain and two acidic regions
Source: BMC Cell Biol. 2010 Dec 31;11:101. doi: 10.1186/1471-2121-11-101 (PMC3022720; doi:10.1186/1471-2121-11-101)
Supplement: Additional file 1 — Real-Time PCR analysis of ChIP assays. Primers for the Real-Time PCR analysis of ChIP assays (Table S1). Raw data for the y2 ct6 anti-Cp190 ChIP (Table S2); for the myc-CP190dBTB anti-Cp190 ChIP (Table S3); for the CP190dC(En15) anti-Cp190 ChIP (S4). [file 1471-2121-11-101-S1.DOC]

**TABLE S1 Primers for Real-Time PCR**

| Locus | primers | Coordinate | Reference |
| --- | --- | --- | --- |
| gypsy | TTCTCTAAAAAGTATGCAGCACTT  CACGTAATAAGTGTGCGTTGA |  | Kurshakova, M. et al 2007 (6) |
| 1A2 | CCTACTTTTTTGCGAGGGACTTC,  TTGCTGTGACCAAGAGTATGCA | Chr X, 255603-255671 | Bushey, A. M. et al 2009 |
| 1A6 | TCTACCTGTTGCATTATTCTCC,  GCCTTTTAAGGTTACCTATTACAG | Chr X, 266202..266572 | Kurshakova, M. et al 2007 (6) |
| 62D | TGATACCAGGCGAACAGAAATC,  TTTGGGCTTGGTGAGAACAG | Chr 3L, 2244127..2244275 | Kurshakova, M. et al 2007 (6) |
| Fab-8 | CAAGCTGTGCAAGGCATTTG,  TTCGAAGCAGAGAATGGAACTCT | Chr 3R, 12744839..12744898 | Bushey, A. M. et al 2009 (2) |
| CTCF2 | CCAGCCCCTTTGCAGATTT,  CGTAACGTTGCTCCAAGTTTGTT | Chr X, 2705284..2705352 | Bushey, A. M. et al 2009 (2) |
| CTCF12 | CTCCGGTTTCGTGTTGCAA  GGGCACTCGGTGATTCTAACTG | Chr 3R, 22692692..22692755 | Bushey, A. M. et al 2009 (2) |
| CTCF13 | GTTTTACAGGCACCCCCACAT  TAGCGAGTGGCGCCATCTA | Chr 3R, 24238375..24238434 | Bushey, A. M. et al 2009 (2) |
| BXC100/Fab-6 | GCAGTTGCAGCTGAAGGATA,  TGGGAGATACAAAGATACCCAGA | Chr 3R, 12708493..12708592 | Holohan, E. E. et al 2007 (4) |
| BXC114/mcp | GCGTGAGAGTAAGTGAGACAACAG,  TAAGGAGGAAGACTACATCAATAA | Chr 3R, 12695186..12695235 | Holohan, E. E. et al 2007 (4) |
| SCS’ | CGATATTCTTCAACCAACCGA,  TTCGTTTGAATTGTGAAGCG | Chr 3R, 7788814..7788895 | Bushey, A. M. et al 2009 (2) |
| BEAF-A2 | ACCGCATTTAGGCAGCAGTT,  GCGACTTTACATTGTGAGGACAA | Chr 2L, 18688688..18688773 | Jiang, N. et al 2009 (5) |
| BEAF-A3 | ATCTTAGAAACCCGGCGAGCAG  TGAGCAAGATAGGCACGAAAGTCC | Chr X, 690427-690573 | Jiang, N. et al 2009 (5) |
| BEAF-AB3 | CGAAGCGAGCCGAACTCATTTT  AGAACTGCGAACATTCATAATT | Chr 3R, 7793051..7793292 | Jiang, N. et al 2009 (5) |
| BEAF-B12 | GTGACCAGATCTCGAGACAGTAA TTAAACTATCGATGCGTATTCAGA | Chr 2L, 17477866..17478009 | Jiang, N. et al 2009 (5) |
| BEAF-B13 | GTCGTTCGATATAATACCACACAA  CCATTTGGTATGAAACGCTGTA | Chr 3R, 6522157..6522336 | Jiang, N. et al 2009 (5) |
| BEAF-B16 | ATATCATCAGCTGTCCCAACCATC  ACGTGGCGTTTTGTTCGTCTG | Chr 2L, 10517317..10517488 | Jiang, N. et al 2009 (5) |

**TABLE S2 *y^2^ ct^6^* anti-Cp190 ChIP**

| Locus | Anti-CP190 ChIP:Input (normalized to Fab8) | S.D. | Pre-Immune ChIP:Input | S.D. |
| --- | --- | --- | --- | --- |
| Gypsy | 1.925408 | 0.38776 | 0.001592 | 0.000528 |
| 1A2 | 0.560124 | 0.00898 | 0.001683 | 0.000554 |
| 1A6 | 0.002158 | 0.001161 | 0.004317 | 0.004736 |
| 62D | 0.721294 | 0.103769 | 0.010242 | 0.018672 |
| Fab-8 | 1.003169 | 0.030141 | 0.002948 | 0.002711 |
| CTCF-2 | 0.044663 | 0.007261 | 0.00238 | 0.001138 |
| CTCF-12 | 0.981041 | 0.054443 | 0.002408 | 0.001622 |
| CTCF-13 | 0.053057 | 0.007314 | 0.00435 | 0.00249 |
| BXC-100 | 0.393605 | 0.025272 | 0.003892 | 0.002371 |
| BXC-114 | 0.540218 | 0.077832 | 0.008549 | 0.00542 |
| SCS` | 0.768625 | 0.057313 | 0.000702 | 0.000325 |
| BEAF-A2 | 0.292903 | 0.065878 | 0.00083 | 0.000202 |
| BEAF-A3 | 0.010943 | 0.010999 | 0.000302 | 0.000143 |
| BEAF-AB3 | 0.015141 | 0.002183 | 0.000254 | 0.000117 |
| BEAF-B12 | 0.041934 | 0.008587 | 0.000765 | 0.000199 |
| BEAF-B13 | 0.010061 | 0.004493 | 0.000709 | 2.65E-09 |
| BEAF-B16 | 0.273744 | 0.038778 | 0.000555 | 0.000611 |

**TABLE S3 myc-CP190dBTB anti-Cp190 ChIP**

| Locus | Anti-CP190 ChIP:Input (normalized to Fab8) | S.D. | Pre-Immune ChIP:Input | S.D. |
| --- | --- | --- | --- | --- |
| Gypsy | 0.288269 | 0.010088 | 0.132376 | 0.033037 |
| 1A2 | 0.200601 | 0.039931 | 0.057568 | 0.00186 |
| 1A6 | 0.074325 | 0.084679 | 0.008158 | 0.00024 |
| 62D | 0.113351 | 0.02153 | 0.023552 | 0.370236 |
| Fab-8 | 1.003928 | 0.049948 | 0.043306 | 0.011388 |
| CTCF-12 | 0.495325 | 0.065529 | 0.064958 | 0.014904 |
| BXC-100 | 0.791924 | 0.097928 | 0.064233 | 0.016628 |
| BXC-114 | 1.838592 | 0.019606 | 0.04632615 | 0.002879452 |
| SCS` | 2.485015 | 0.015318 | 0.102761 | 0.046588 |
| BEAF-A2 | 1.992076 | 0.004611 | 0.10449 | 0.033677 |
| BEAF-B16 | 1.507753 | 0.706544 | 0.092772 | 0.037254 |

**TABLE S4 CP190dC(En15) ChIP**

| Locus | Anti-CP190 ChIP:Input (normalized to Fab8) | S.D. | No antibody control ChIP:Input | S.D. |
| --- | --- | --- | --- | --- |
| Gypsy | 1.933329 | 0.233264 | 0.040914 | 0.010287 |
| 1A2 | 2.164275 | 0.315891 | 0.018536 | 0.011665 |
| 1A6 | 0.122084 | 0.038194 | 0.003534 | 0.001695 |
| 62D | 2.859943 | 0.348904 | 0.008412 | 0.012409 |
| Fab-8 | 1.003276 | 0.033605 | 0.021695 | 0.00714 |
| CTCF-12 | 1.04371 | 0.115364 | 0.021944 | 0.007241 |
| BXC-100 | 1.053593 | 0.137179 | 0.028179 | 0.008539 |
| BXC-114 | 0.898194 | 0.097203 | 0.018197272 | 0.006895777 |
| SCS` | 0.974603 | 0.000283 | 0.042024 | 0.021539 |
| BEAF-A2 | 0.988925 | 0.001784 | 0.041394 | 0.02262 |
| BEAF-B16 | 0.50206 | 0.002512 | 0.028231 | 0.012362 |

**REFERENCES**

1. Akbari, O. S., D. Oliver, K. Eyer, and C. Y. Pai. 2009. An Entry/Gateway cloning system for general expression of genes with molecular tags in Drosophila melanogaster. BMC Cell Biol 10:8.

**2. Bushey, A. M., E. Ramos, and V. G. Corces. 2009. Three subclasses of a Drosophila insulator show distinct and cell type-specific genomic distributions. Genes Dev 23:1338-50.**

**3. Butcher, R. D., S. Chodagam, R. Basto, J. G. Wakefield, D. S. Henderson, J. W. Raff, and W. G. Whitfield. 2004. The Drosophila centrosome-associated protein CP190 is essential for viability but not for cell division. J Cell Sci 117:1191-9.**

**4. Holohan, E. E., C. Kwong, B. Adryan, M. Bartkuhn, M. Herold, R. Renkawitz, S. Russell, and R. White. 2007. CTCF genomic binding sites in Drosophila and the organisation of the bithorax complex. PLoS Genet 3:e112.**

**5. Jiang, N., E. Emberly, O. Cuvier, and C. M. Hart. 2009. Genome-wide mapping of boundary element-associated factor (BEAF) binding sites in Drosophila melanogaster links BEAF to transcription. Mol Cell Biol 29:3556-68.**

**6. Kurshakova, M., O. Maksimenko, A. Golovnin, M. Pulina, S. Georgieva, P. Georgiev, and A. Krasnov. 2007. Evolutionarily conserved E(y)2/Sus1 protein is essential for the barrier activity of Su(Hw)-dependent insulators in Drosophila. Mol Cell 27:332-8.**
